# Upper elementary students’ self-efficacy, affect, and avoidance associated with multisyllabic words: an exploratory study

**DOI:** 10.3389/fpsyg.2025.1562958

**Published:** 2025-08-05

**Authors:** Zoi A. Traga Philippakos, Margaret Quinn, Adalea Davis

**Affiliations:** ^1^Theory and Practice in Teacher Education, The University of Tennessee, Knoxville, Knoxville, TN, United States; ^2^Teaching, Learning, and Culture, Texas A&M University, College Station, TX, United States

**Keywords:** an exploratory study self-efficacy, Affect, avoidance, reading, big words, multisyllabic, Decoding, spelling upper elementary

## Abstract

**Introduction:**

The purpose of this study was to develop and validate a questionnaire that addressed reading and spelling of big words to understand upper elementary learners’ perceptions of their abilities and challenges in relation to multisyllabic words.

**Methods:**

The development of this questionnaire was part of a larger research project that aimed to develop instructional resources for upper elementary learners. Participants were 108 students across grades 3 to 5.

**Results:**

The results of an Exploratory Factor Analysis (EFA) found three factors that addressed self-efficacy for processes and tasks, affect, and avoidance. All factors correlated with reading measures with the exception of avoidance. Further, differences on self-efficacy and affect were found between the lower and low-average reading group. Finally, growth was found on self-efficacy and affect toward reading of big words as a result of instruction. Limitations and implications are discussed.

## Introduction

In upper elementary grades, students transition from learning to read to reading to learn (Kamil et al., 2008; Stevens et al., 2022). This transition results in the use of texts that consist of longer sentences, such as complex and compound sentences, as well as longer paragraphs. There are significant working memory and cognitive demands on learners who need to make meaning while processing complex ideas (Wijekumar et al., 2017). However, this complexity for some students is not solely due to the structure of the sentences and length of the texts, but also arises from the composition of the included words. As upper elementary learners encounter more advanced texts, the words within those texts progressively increase in length and complexity. For students who find the decoding process of single-syllable words challenging, the decoding of multisyllabic words is far more challenging as such words are difficult to decode when utilizing strategies typical to monosyllabic words, and also morphemes within these long words carry meaning. Undoubtedly, when students lack knowledge of decoding strategies, of affixes, and roots, their abilities to decipher word and meanings are hindered, and they are unable to add to their knowledge base. Additionally, challenges at the word level impact their self-efficacy in decoding such words, increasing both their apprehension toward decoding and their affect (see Traga Philippakos et al., 2024; Traga Philippakos et al., In Press).

The purpose of this study is to develop and validate a questionnaire that addressed reading and spelling of big words to more deeply understand upper elementary learners’ perceptions of their abilities and challenges associated with multisyllabic words. The development of this questionnaire was part of a larger research project that aimed to develop instructional resources for upper elementary learners. Research on overall reading and specific motivation associated with reading and spelling big words, including relevant questionnaires and findings, is presented to demonstrate the *a priori* theoretical basis on which the developed instrument, presented in this study, was built. The terms survey, scale, and questionnaire are used interchangeably, but for the purposes of this study, we will refer to the tool we developed as Questionnaire.

### Reading and motivation

Struggling readers usually have little motivation (Boardman et al., 2008), which in turn leads to worsened comprehension and strategy usage (Morgan and Fuchs, 2007; Guthrie and Wigfield, 2000). Guthrie and Humenick (2004) found students’ motivation to read a text was influenced by factors such as autonomy, interest, perceived value, and social opportunities relating to the text. The authors suggest that teachers can enhance student motivation by emphasizing the significance of the content knowledge inherent in the text. That said, Boardman et al. (2008) claimed that motivational belief instruction must be paired with a skill- and strategy-focused comprehensive program in order to be effective. Relatedly, Saqui et al. (2019) created a questionnaire adapted from the Children’s Intervention Rating Profile (Witt & Elliott) to understand their motivation to read big words associated with social validity. It included 7 items focusing on the children’s enjoyment of, evaluation of, and improvement from the multisyllabic word reading intervention. This instrument provides important initial information regarding students’ perceptions of their own experiences with reading and spelling big words, but is limited in terms of its scope.

Toste et al. (2017) explored the impacts of a multisyllabic word reading intervention by including motivational beliefs training for one subgroup. The students who participated in the reading intervention, which were drawn from a sample of 3^rd^ and 4^th^ grade struggling readers, outperformed the students who participated in the business-as-usual condition. The findings revealed that students who received additional motivational-beliefs training outperformed those who received only the reading intervention in sentence-level comprehension.

This research suggests important connections between motivations and beliefs and reading success and intervention uptake. Following this work, Toste et al. (2019), replicated this study among 4^th^ and 5^th^ grader struggling readers. While there did not appear to be differences in intervention groups based on the inclusion or lack of motivational training, the researchers found that students who participated in the intervention had lower self-concept following the intervention than those who had participated in business as usual, suggesting that, perhaps, once students participated in the intervention they grew in their self-awareness and were better equipped to evaluate themselves more realistically. In this study, the researchers used the Reading Self-Concept Scale (Chapman and Tunmer, 1995), which features 30 items focused on competence, difficulty, and attitude but measures self-concept towards reading more broadly than just focusing on students’ experiences with multisyllabic words.

Research examining students’ self-efficacy and affect towards reading and spelling big words is relatively limited. Given the frequency and preponderance of upper elementary students’ encounters with multisyllabic words, it is important to develop tools to better understand their perceptions and experiences. Further, it would be beneficial for teachers to assess their students’ self-efficacy and perspectives on tasks before delivering instruction. That way, first, teachers could instructionally address not only the content but also students’ motivation. Second, it is important for teachers to assess whether their instruction leads to change in students’ self-efficacy and emotional responses. Evaluations of this nature could help establish clearer instructional goals and provide additional supports to students. Finally, researchers can further examine connections among constructs associated with decoding and encoding of multisyllabic words and their impact on comprehension and other reading components. As a result, an investigation of this nature has implications for both research and practice.

### Present study

This study reports on an Exploratory Factor Analysis (EFA) on a scale designed to measure self-efficacy associated with reading and spelling multisyllabic words. We conducted an EFA instead of a Confirmatory Factor Analysis (CFA) since we did not have a predefined factor structure (Costello and Osborne, 2005); thus, we could not test with a CFA the goodness-of-fit of our model (Kline, 2015) as our goal was to examine the underlying dimensions or factors in our present dataset (Brown, 2015; Fabrigar et al., 1999). The study contributes to the field as it attempts to investigate learners’ self-efficacy for reading and spelling multisyllabic words and does not examine students’ self-efficacy about reading skills or reading as a practice in general. In addition, this study examines self-efficacy and affect for upper-elementary learners who are academically progressing from foundational skills to multisyllabic reading, spelling, and composing in English Language Arts and in the disciplines (e.g., science). The self-efficacy scale included items that measured processes, avoidance, goals, and affect. In addition to the examination of the construct dimensions through factorial analysis, we also examined validity through correlations among the factors and correlations to reading achievement.

## Methods

### Participants and setting

Participants were 108 learners across grades 3 to 5 (*n* = 54 female learners). Students were in their majority White/Caucasian (93.51%) (see Table 1 with demographics information). The school is located in a rural area in the southeast region of the United States and is a Title I school serving a total of 244 students (*n* = 118 female) across Pre-Kindergarten to Grade 5. All participants qualified for free/reduced lunch. Grade 3 to 5 learners participated in a larger research project which sought to develop and evaluate a program targeting multisyllabic decoding and encoding (see Traga Philippakos et al., 2024; in press). Parental consents were collected granting participation of learners to the study.

**Table 1:** Demographics Information.

	Female	Male	Black	White	Multiracial
Grade 3	11 (42.30%)	15 (57.69%)	0	22 (84.61%)	4 (15.38%)
Grade 4	23 (56.09%)	18 (43.90%)	0	41 (100%)	0
Grade 5	20 (48.78%)	21 (51.21%)	1 (2.43%)	38 (92.68%)	2 (4.87%)
Total	54 (50%)	54 (50%)	1 (0.92%)	101 (93.51%)	6 (5.55%)

### Procedures

The self-efficacy scale was administered in the classroom by research personnel, and students completed the measure on paper. Practice items were modeled by the administrator to explain the meaning of confidence (how certain you are about something; 100% sure; 50% sure, 0%) and the meaning of affect (how much you like doing something). The administrator explained how different individuals may have different percent confidence when completing tasks, and that this was expected and as they were all different people with different experiences, gifts, and abilities. After the practice items, the administrator read each of the sentences out loud and asked students to independently select and mark with a circle the number that best supported their confidence/certainty or affect toward specific tasks. The decision to read the items out loud to students was made to remove any decoding challenges learners might have possibly faced and to assure the timely completion of the scale by all learners since the administrator directed the pace for the completion of the task. Students were directed to respond with honestly and the practice items earlier further highlighted the importance of responding honestly and the realization that “it is okay” for responses to be different and not the same among participants.

The scale was administered before students completed any of the reading assessments so their responses would not be affected by the specific tasks. After completion of the self-efficacy questionnaire, students logged on their computers and completed the CAPTI subtests of word recognition, morphology, vocabulary, reading efficiency, sentence processing, and reading comprehension (see: Measures below).

The larger study of which this research was a part utilized design-based research (Philippakos et al., 2021; Reinking and Bradley, 2008) to examine the efficacy of an intervention targeting multisyllabic word reading and spelling over two cycles of implementation. In the first cycle, within each grade, one classroom received the instruction associated with the intervention; the other class in that grade level received instruction later, as a part of a second cycle of implementation of design-based research (Traga Philippakos et al., 2023). At the beginning and end of the first cycle, learners were assessed on their motivation using the same scale and on the reading measures.

### Measures

#### Scale and item development

Based on the authors’ previous work (e.g., MacArthur et al., 2016; Traga Philippakos, 2019; Traga Philippakos et al., 2023; Traga Philippakos, 2020), a Likert-scale was used with a range of 0 to 100. If a student selected 100% on the scale, that meant that they were confident and sure, while a selection of 0% would imply the opposite, or 50% that they were neither strongly confident/sure or unconfident/unsure. There were choices in between and reference to percentages.

Regarding item development, the researchers developed a list of items that addressed efficacy for reading, spelling, understanding, and writing two-syllable words, words with prefixes, words with suffixes (e.g., I can read big words with three syllables), avoidance (I usually avoid reading big words), and self-regulation (e.g., I can reread words to check their spelling). The items on affect were modeled on previous scales that addressed affect for writing (Traga Philippakos, 2019) and reading (McKenna and Kear, 1990) (e.g., I really enjoy reading big words in sentences).

Once the items were developed by the researchers (a total of 52 items), they were shared with a group of teachers across grades 3 to 5 (*n* = 9 teachers; three per grade level) who were asked to sort the items across the categories we had initially developed and to add items to a category we named “unclear” if teachers felt unsure about an item’s classification. For the items teachers considered unclear, we asked them to explain what affected (from their point of view) the item’s clarity. The teachers completed this knowing that their feedback was solicited to better examine ways to examine the self-efficacy of students in their grades regarding the reading, spelling, understanding, writing, avoiding, and liking big words. Teachers identified 15 items as challenging to understand (unclear) because the language was too complex for their students as phrased (two items) or the item needed an example to be clear (10 items) or the item included two parts (two-part question) (three items), and, consequently, it was unclear what it intended to measure. Based on this feedback, we revised overall 15 items and eliminated 12 items resulting to a total of 30 final items. We then shared the scale with two researchers who had been conducting relevant research in motivation for feedback. The researcher supported further the refinement of the items as they were asked to sort them and score each item on clarity and specificity to what it intended to measure. The scale for clarity and specificity ranged from 0 (not clear) to 2 (very clear). The reliability of the researchers by item was high for processes, affect, and avoidance (ICC = 0.98, 0.99, and 0.99 respectively) and adequate for self-regulation (ICC = 0.82). Discrepancies between raters were discussed and resolved; based on the feedback, revisions were made mostly on the phrasing of the items and on their grammatical presentation; no items were eliminated. The final scale included a total of 30 items. There were items referring to strategies (4), reading (7), spelling (6), writing (2), understanding (3), avoidance (2), self-regulation (2) and affect (4).

#### CAPTI/Read Basix

The CAPTI/Read Basix is a web-based reading assessment which students complete on their electronic devices using a unique login and password created by the administrator. In this case the main administrator was the curriculum coordinator who uploaded classroom rosters and developed their access code with consultation by the company when needed. The subtests included are word recognition and decoding, vocabulary, morphology, sentence processing, reading efficiency, and reading comprehension. The subtests have demonstrated acceptable to strong reliability coefficients (ranging from 0.674 to 0.927; Sabatini et al., 2019). Within each subtest, students can complete or skip practice items and then they proceed with the actual test-items. Table 1 presents students’ reading performance by grade.

#### Gray oral reading test—5^th^ edition (GORT-5)

GORT-5 (Wiederholt and Bryant, 2012) is a standardized test that examines reading rate, accuracy, fluency, and comprehension. The GORT-5 has high internal consistency (alphas > 0.90), test–retest reliability (alphas > 0.85) and it is sensitive to reading difficulties (sensitivity = 0.82, low levels of false positives). The test is administered individually and asks students to read passages fluently and answer comprehension questions.

#### Test of silent reading efficiency and comprehension (TOSREC)

The TOSREC (Wagner et al., 2010) measures learners’ silent reading comprehension and can be group-administered. Students are asked to silently read and evaluate the truthfulness of sentences within three minutes. The TOSREC has strong reliability (> 0.85 across all grade level tests).

#### Test of word reading efficiency-2nd edition (TOWRE-2)

The TOWRE-2 (Torgesen et al., 2012) includes two subtests: the Sight Word Efficiency test and the Phonemic Decoding Efficiency. The former measures accurate, timed high-frequency word reading and the latter accurate, timed pseudoword decoding. Each measure allots 45 s for students to read a list of words increasing in complexity. The TOWRE-2 is individually administered. The measure is reliable in measuring reading efficiency (alphas > 0.90).

These reading measures were used because of their reliability and validity; further, they are measures that are commonly used in reading research. Finally, we chose to use CAPTI because it was computerized, and we were able to collect information on subscales that address word reading, morphology and comprehension in relatively short time (in relation to the rest of the measures).

### Analysis

We conducted an Exploratory Factor Analysis (EFA) using principal component factor analysis (PCA) with Varimax rotation including all items because we wanted to examine whether the constructs would be differentiated. Thus, we chose not to run the affect items separately, but rather we wanted to examine the scale overall as a measure of motivation. PCA was selected as PCA transforms a large set of observed variables into a smaller set of uncorrelated components, which can simplify complex data and reduce dimensionality while retaining as much variance as possible (Fabrigar et al., 1999). This makes it a practical choice in the early stages of developing a tool, especially when we aim to identify key components of a construct without assuming a specific underlying structure (Costello and Osborne, 2005). PCA is exploratory and our goal was to explore the structure in our dataset since we did not have a clear expectation on this structure (thus, the EFA) (Field, 2013). Also, while PCA components do not necessarily represent latent variables in the same way that methods like PAF or Maximum Likelihood Estimation (MLE) do, they can still identify dimensions that explain the majority of variance in the data (Tabachnick and Fidell, 2013). We also used PCA as when combined with varimax rotation which maximizes the variance of each factor, PCA provides a straightforward way to interpret the resulting components (Costello and Osborne, 2005; Kaiser, 1958). It also does not require assumptions about the relationships between observed variables and unobservable factors. This makes it robust when working in the beginning stages of tool development like we did in this study (Tabachnick and Fidell, 2013). Assumptions were examined and met. Specifically, the Kaiser-Meyer-Olkin (KMO) index, which is a measure of sampling adequacy was 0.918 and exceeded the recommended value of 0.6 (Kaiser, 1970). The Bartlett’s Test of Sphericity (Bartlett, 1954) was statistically significant (*χ*^2^ = 1954.78, *p* < 001) indicating that the data were appropriate for a factor analysis. Small coefficients with a value of < 0.40 were suppressed using guidelines for conducting EFA (see Field, 2009). Subsequently, for validation purposes, we examined correlations between the factors and the reading subtests of CAPTI and the measures of TOWRE, TOSREC, and GORT. Finally, we examined the scale’s sensitivity to change by examining differences on self-efficacy, affect, and avoidance after instruction was provided.

## Results

The initial analysis revealed five factors and items intended to measure understanding and self-regulation cross loading with reading/spelling items for two or three syllable words and strategy items. Three factors were extracted after the examination of the scree plot and of eigenvalues >1. Items 8 and 10 did not load and were removed. Items 25 and 26 that addressed self-regulation were removed as one of them did not load and the other loaded under enjoyment with low coefficient (0.401). The analysis resulted in three factors where processes and strategies for reading and spelling formed the first factor (alpha = 0.94) with 20 items and explained 46.488% of the variance; the second factor addressed affect, included five items, and explained 9.266% of the variance (alpha = 0.87); the last factor included two items that measured avoidance and explained 5.728% of the variance (alpha = 0.62). Cumulatively, the three factors (processes and strategies, affect, and avoidance) explained 61.353% of the variance. Information pertaining to the EFA is presented in Table 2.

**Table 2:** Rotated component matrix.

	Factors		
Questionnaire Items	Self-Efficacy Tasks/Processes for reading and spelling	Affect	Avoidance
1	0.716		
2	0.853		
3	0.807		
4	0.753		
5	0.818		
6	0.797		
7	0.757		
9	0.801		
11	0.514		
12	0.787		
13	0.745		
14	0.811		
15	0.792		
16	0.743		
17	0.692		
18	0.625		
27	0.469		
29	0.558		
30	0.554		
31	0.563		
19		0.796	
21		0.778	
23		0.846	
24		0.826	
20			0.830
22			0.843
Eigenvalue	12.087	2.409	1.489
% of var.	46.488	9.266	5.728
Cumulative %	46.488	55.754	61.489

### Correlations with reading achievement

To further examine the construct validity of the factors, correlations were explored among the factors and between the factors and reading subtests of CAPTI (six subtests total), GORT-5, TOSREC, and TOWRE-2. The reading measures were highly correlated with one another and with the processes and strategies subscale, while affect was significantly correlated with processes and strategies (*p*s < 0.01). Overall, avoidance did not correlate with the rest of the scales or with the reading measures. Correlational analyses are presented in Table 3.

**Table 3:** Correlations between reading measures and motivation constructs.

	Tasks Processes	Affect	Avoidance	SWE Scaled Score	PDE Scaled Score	TOWRE Scaled Score	RAW_TOSREC	TOSREC index	Scaled Rate GORT	Scaled Accuracy GORT	Scaled Fluency GORT	Scaled ComprehGORT	Sum scaled GORT	Word Recogn	Vocab/ry	Morph/gy	Sentence Processing	Reading Efficien
Tasks Processes																		
Affect	0.484^**^																	
Avoidance	0.074	−0.033																
SWE Scaled Score	0.526^**^	0.275^**^	0.022															
PDE Scaled Score	0.541^**^	0.279^**^	−0.112	0.835^**^														
TOWRE Scaled	0.550^**^	0.295^**^	−0.038	0.933^**^	0.940^**^													
RAW_TOSREC	0.527^**^	0.182	−0.054	0.772^**^	0.737^**^	0.775^**^												
TOSREC index	0.454^**^	0.163	−0.095	0.722^**^	0.694^**^	0.727^**^	0.973^**^											
SCALED_RATE	0.582^**^	0.284^**^	−0.020	0.876^**^	0.803^**^	0.856^**^	0.803^**^	0.737^**^										
Scaled_Accuracy_GORT	0.542^**^	0.255^**^	0.033	0.800^**^	0.806^**^	0.837^**^	0.718^**^	0.666^**^	0.841^**^									
Scaled Fluency_GORT	0.578^**^	0.297^**^	−0.026	0.861^**^	0.837^**^	0.876^**^	0.760^**^	0.730^**^	0.927^**^	0.958^**^								
Scaled Comprehension_GORT	0.389^**^	0.229^*^	0.004	0.494^**^	0.456^**^	0.482^**^	0.449^**^	0.393^**^	0.604^**^	0.645^**^	0.650^**^							
Sum_scaled_GORT	0.535^**^	0.290^**^	−0.013	0.752^**^	0.718^**^	0.754^**^	0.677^**^	0.636^**^	0.848^**^	0.888^**^	0.914^**^	0.902^**^						
Word_Recognitoion_	0.426^**^	0.127	−0.124	0.612^**^	0.700^**^	0.655^**^	0.623^**^	0.565^**^	0.658^**^	0.644^**^	0.684^**^	0.539^**^	0.674^**^					
Vocabulary	0.497^**^	0.016	−0.081	0.577^**^	0.607^**^	0.585^**^	0.608^**^	0.562^**^	0.609^**^	0.558^**^	0.600^**^	0.508^**^	0.609^**^	0.788^**^				
Morphology_	0.483^**^	0.054	−0.015	0.578^**^	0.708^**^	0.665^**^	0.586^**^	0.539^**^	0.631^**^	0.651^**^	0.667^**^	0.517^**^	0.653^**^	0.784^**^	0.836^**^			
Sentence Processing_	0.487^**^	0.138	0.014	0.554^**^	0.629^**^	0.600^**^	0.603^**^	0.536^**^	0.628^**^	0.557^**^	0.598^**^	0.471^**^	0.590^**^	0.702^**^	0.774^**^	0.805^**^		
Reading Efficiency_	0.548^**^	0.067	−0.098	0.494^**^	0.615^**^	0.560^**^	0.604^**^	0.543^**^	0.599^**^	0.613^**^	0.625^**^	0.543^**^	0.649^**^	0.720^**^	0.789^**^	0.790^**^	0.729^**^	
Reading Comprehension_	0.391^**^	0.083	−0.143	0.274^**^	0.395^**^	0.354^**^	0.408^**^	0.446^**^	0.353^**^	0.440^**^	0.439^**^	0.461^**^	0.496^**^	0.572^**^	0.619^**^	0.559^**^	0.427^**^	0.705^**^

** Correlation is significant at the 0.01 level.

* Is significant at the 0.005 level.

### Differences by level and grade

To examine the validity of the factors we examined whether there were detectable differences based on students’ reading ability and grade level. Using the guidelines from CAPTI word recognition, we classified learners using their scaled score as either weak in word recognition (range 190–235) or low average (236–249). The results of multiple analysis of variance found statistically significant differences by level [*F*(1, 98) = 18.95, *p* < 0.001] with learners who were in the weak range (M = 44.12; SD = 24.02) having lower self-efficacy on processes and strategies for multisyllabic reading and spelling compared to those who were in the low-average range (M = 66.28; SD = 25.83) (*d* = 0.88). There was no statistically significant difference for avoidance or affect by word recognition level (*p* > 0.05). To further examine the validity of the factors we compared the scores for students across grades with the Bonferroni correction applied (*p* equal or less than 0.016). Marginally, no statistically significant difference was detected by grade level (*p* = 0.018).

### Change following instruction

In order to examine the measure’s sensitivity, we examined change to responses following exposure to the instructional intervention. Analysis of Covariance was conducted for each subscale using responses at pretest as a covariate. The results for processes and strategies were significant [*F*(1,97) = 69.88, *p* < 0.001] with students who received instruction having higher self-efficacy on processes and strategies (M = 72.91; SD = 20.36) than those who did not (M = 66.77; SD = 23.23) following the intervention (*d* = 0.28). Similarly, there were statistically significant differences for affect [*F*(1,97) = 33.495, *p* < 0.001] with learners who received instruction indicating higher affect (M = 56.11; SD = 23.98) toward reading and spelling of words than students who were did not receive relevant instruction (43.93; SD = 21.90) (*d* = 0.58). Finally, no statistically significant differences were found on avoidance [*F*(1, 97) = 4.094, *p* = 0.46] with students who received instruction (M = 39.06; SD = 37.05) and those who did not (M = 39.89; SD = 35.85) having no substantive change in their avoidance toward reading and spelling multisyllabic words. Examination of differences by grade and gender across the two groups using pretest as a covariate did not detect statistical differences (*p* > 0.05). See Table 4 for means and standard deviations.

**Table 4:** Results by gender, grade, and effects of instruction.

	Pre_Processes and Tasks	Post_Processes and Tasks	Pre_Affect	Post_Affect	Pre_Avoidance	Post_Avoidance
Female	50.62 (25.19)	70.54 (21.00)	45.38 (30.78)	51.58 (22.40)	38.79 (30.32)	34.69 (33.97)
Male	56.82 (27.97)	69.06 (23.10)	49.16 (33.68)	48.31 (24.96)	40.97 (31.23)	44.37 (38/19)
Grade 3	50.86 (23.26)	67.29 (21.01)	61.68 (33.38)	59.94 (15.72)	44.03 (31.96)	28.63 (28.41)
Grade 4	48.92 (27.10)	69.23 (20.45)	45.71 (31.89)	47.37 (22.96)	33.53 (28.79)	44.45 (38.79)
Grade 5	60.35 (27.51)	71.82 (24.21)	39.69 (29.30)	46.71 (26.83)	43.59 (31.30)	40.92 (37.32)
Group 1	48.37 (26.31)	72.91 (20.36)	45.89 (33.47)	56.11 (23.98)	41.12 (34.25)	39.06 (37.05)
Group 2	58.51 (26.30)	66.77 (23.23)	48.51 (26.30)	21.90 (49.96)	38.77 (27.30)	39.89 (35.85)

M, mean; SD, standard deviation.

## Discussion

The purpose of the study was to develop and validate a measure of motivation toward multisyllabic decoding and encoding for upper elementary learners. The results showed that the developed measure includes the constructs of self-efficacy for processes and tasks and processes, affect, and avoidance. Further, the results showed that at least two of the subscales highly and significantly correlate with one another and with reading measures while they also can differentiate among students’ reading performance. Finally, the results show that the factors are sensitive to change due to learning on multisyllabic decoding and encoding. Considering previous research, the findings of the current study will be discussed with attention to (1) the ways in which the developed measure can effectively address multisyllabic word encoding and decoding self-efficacy and motivation for upper elementary students, (2) the use of such a tool to better understand students’ experiences with reading broadly and as a result of targeted intervention, and (3) the research and practical implications of the developed instrument.

### Aspects of multisyllabic self-efficacy

Research has long considered students’ motivation for reading and their self-efficacy and ability beliefs towards reading. Foundational research as well as more recent work has determined approaches to measuring self-efficacy and motivation, and also found important linkages between such factors and students’ ability and engagement (e.g., Carroll and Fox, 2017; McGeown et al., 2016; Schunk and Rice, 1993; Solheim, 2011; Wigfield, 1997). In these studies, students’ perceived self-efficacy and their motivation to read was related to reading subcomponents such as comprehension and/or word reading, among others. Critically, studies have provided myriad approaches to gathering students’ self-efficacy and motivation with a number of tools, instruments, and methods to better understand these constructs within students. For example, Davis et al. (2018) comprehensively reviewed 16 different scales operationalizing reading motivation detailing the development of such tools, focal constructs, psychometrics, and strengths and challenges associated with each. Across these measures, Davis et al. (2018) determined that motivation instruments consistently reported reliability information but were less consistent with robust information around validity. Further, they note theoretical issues in the scales that have been addressed elsewhere as well (Conradi et al., 2014).

As it relates to self-efficacy, research suggests variable approaches for measurement as well as theoretical discrepancies in terms of the relationship between self-efficacy and motivation. For some, self-efficacy is a facet of motivation or inextricably linked to motivation, often related to intrinsic motivational factors (e.g., Wigfield, 1997); while for others, it is a separate construct (e.g., Hedges and Gable, 2016). Research has long sought to measure motivation and self-efficacy by prompting students to consider their affect and feelings around reading and spelling, their perceived abilities, intrinsic and extrinsic purposes for reading, feelings towards different types of reading (e.g., academic vs. leisure reading), and more (Gambrell et al., 1996; Guthrie et al., 2004; Lee and Jonson-Reid, 2016; McKenna et al., 1995; Wigfield and Guthrie, 1997, among many others). Many instruments detailed in the literature examine a combination of factors related to motivation and self-efficacy, however, the relationships between these factors are not well elucidated (Davis et al., 2018). The current study adds to literature by examining the constructs of self-efficacy and to an extent, motivation, but importantly, within the specific context of multisyllabic word reading and spelling. Measures previously used and detailed in the research typically examine students’ broad-based experiences with reading holistically, rather than with a specific part of reading, subskill, and context (e.g., students’ feelings about their encounters with multisyllabic words). Because multisyllabic word reading requires slightly different skills than monosyllabic decoding and encoding (e.g., Enderby et al., 2021; Heggie and Wade-Woolley, 2017) and it likely presents a considerable challenge to students who are struggling with reading and writing in the upper elementary grades, examining self-efficacy and motivational factors related to this specific context of multisyllabic words provides more precise information than a general measure.

### Connections to related factors

As noted, the processes and tasks subscale and the affect subscale were highly correlated to one another. This connection could potentially suggest an overarching motivation construct in which students’ self-efficacy around tasks and processes involved with reading and spelling big words and their feelings towards this act are contributing to the same overall motivational factor. However, additional research would be needed to confirm this. Further, the correlation between processes/tasks and affect suggests that emotional aspects of reading, such as enjoyment or liking are linked to the strategies and processes that individuals use when reading. This relationship is consistent with theories of self-regulated learning and reading motivation, which propose that a reader’s emotional engagement can influence their cognitive strategies and overall reading success (Schunk and Zimmerman, 2012). For instance, a reader who enjoys reading may be more likely to use effective reading strategies, which can, in turn, enhance their reading fluency and comprehension. This is mirrored in research documenting the connections between self-efficacy and affective reactions within the context of reading more broadly (e.g., Linnenbrink and Pintrich, 2003; Walker, 2003). The reading measures were also correlated with each other, which would be expected since reading measures like CAPTI, GORT-5, TOSREC, and TOWRE-2 are designed to assess different facets of reading, and it would be reasonable to assume that these constructs are related. For example, reading fluency (measured by tools like GORT-5 and TOWRE-2) might be strongly related to comprehension (measured by CAPTI or TOSREC), as these processes often work together in reading tasks. The strong correlation among these measures and the processes and tasks subscale on multisyllabic words further supports the idea that high self-efficacy for processes and tasks for multisyllabic reading and spelling connects with comprehension, and fluency and the ability to perform well on those. This provides evidence that the factor is tapping into meaningful constructs related to reading performance.

The apparent lack of a relationship between avoidance and other subscales is worth considering. The expectation might be that those with more positive affect and higher levels of self-efficacy associated with tasks and processes might report less avoidance; however, in the current study, there was no connection positive or negative between avoidance and other self-efficacy and affective factors. It is further possible that a greater number of avoidance-specific items, more nuanced items, and/or more fine-grained tools to explore more negative affective factors may be necessary in order to understand avoidance in this context. In contrast to the current study, research suggests connections between reading/writing work avoidance and other factors such as other aspects of self-efficacy, motivation, and reading attitudes (e.g., Davis et al., 2020) as well as reading achievement (Syal et al., 2024). It should be noted, though, that the lack of significant correlations with the processes/tasks subscale or the reading measures suggests that avoidance may not be directly related to the cognitive aspects of reading performance. This could imply that avoidance operates as a separate construct. It could also indicate that students who avoid reading are not necessarily performing poorly in reading, but may be disengaged for other reasons (e.g., lack of interest, or external factors). It could also be, though, that we only had two items for this factor and additional development and validation is necessary.

In addition to the relations between the subscales, two of the three subscales were correlated to other extant reading measures and were sensitive enough to discern changes to self-efficacy based on participation in a multisyllabic intervention. This study found that students’ multisyllabic tasks and processes responses on the self-efficacy questionnaire were connected to every measure of reading that was utilized in the present inquiry including assessments measuring comprehension, fluency, word reading skills, and more. This further suggests the potential power in exploring students’ feelings of self-efficacy, or lack thereof, in light of the strong correlations to their reading skills. The nature of this correlation, and potential causation or directionality is as yet unknown; however, research suggests that broader, reading-focused self-efficacy is bidirectionally related to reading skills (e.g., Morgan and Fuchs, 2007; Retelsdorf et al., 2014).

In addition to the tasks and processes subscale, which included items around students’ perception of their ability to engage in reading/spelling big words, the affect subscale was related to some reading measures but not as comprehensively as the tasks and processes subscale. As noted in the results, the affect subscale was related to most GORT-4 and TOWRE scores but not CAPTI or TOSREC scores. This may indicate differences in sensitivity in reading measures. The findings of this study parallel others which have found that affective factors broadly associated with reading typically correlate to students’ reading skills (e.g., Malanchini et al., 2017; McGeown et al., 2015). In the present study, the avoidance scale did not correlate with any of the reading measures which again, is worth considering. As noted above, it is possible that because the avoidance items were fewer in number and explained less of the variance in the factor model than the other subscales, they may have lacked overall significance or were not sensitive enough to student difference. Extant literature exploring negative affective factors related to broad reading, i.e., not just multisyllabic, often suggest stronger relations than were uncovered in the present inquiry (e.g., Ho and Guthrie, 2013).

In addition to the subscales relating to one another and to measures of reading, there were important connections to intervention participation. As some students participated in an instructional intervention focused on multisyllabic word reading, an important finding was that students in the treatment group ranked their self-efficacy for tasks and processes as well as their affect significantly higher at posttest than students who had not participated in the intervention. This demonstrates construct and predictive validity, critical for effective measure design. Further, this finding indicates that the developed questionnaire could be a powerful tool in determining response to intervention as it relates to multisyllabic word reading and spelling. In a meta-analysis of intervention research, Unrau et al. (2018) found that reading interventions often yielded increases in both self-efficacy and reading comprehension, and that further, disentangling the impact of the intervention on these constructs independently was difficult. It is important to consider that interventions intending to target skills and support student learning may yield, as showed here, increases in self-efficacy, in addition to skills as measured by various subtests. While avoidance was not different between groups to a statistically significant degree, it is worth noting that avoidance marginally decreased for students in the intervention group, further suggesting that with greater sensitivity, it is possible that avoidance items may, similar to tasks/processes and affect, demonstrate differences between the intervention and control groups in the context of this study or others like it evaluating the impact of interventions focused on multisyllabic words.

### Empirical and practical implications

For students who arrive in upper elementary grades as struggling readers, it is a challenge to catch up (Hernandez, 2011; Scarborough, 2001). As a result, it is likely that low performance will continue and even widen without the provision of interventions. Even though reading measures can detect performance-level challenges, it is important to also consider learners’ self-efficvacy and affect toward not only reading in general but toward decoding and encoding multisyllabic words, particularly given the prevalence of such words as students enter the upper elementary grades (Kearns & Hiebert, 202). This study has implications for research in that it presents important findings associated with a developed self-efficacy instrument that can be utilized to determine affective and motivational factors associated with multisyllabic encoding and decoding and may be able to be used in intervention contexts to determine how students’ self-efficacy may shift. Practically, this study also has implications as such a tool can support teachers in examining the practices they use and whether students’ motivation changes as a result of their instruction. Thus, even though additional validation is needed for this specific tool, teachers could examine students’ self-efficacy and affect before instruction on multisyllabic reading and spelling and after instruction. Initial testing can help identify students who may be apprehensive toward reading and spelling of multisyllabic words and who may need additional motivational support in addition to systematic instruction of strategies for reading and spelling such words. Examination of self-efficacy and affect can inform instructors on the effects of their instruction in supporting students confidence and liking. Also, knowing the relationship of such factors with reading performance, it is important for teachers to instructionally work to support students’ confidence so they can read such words and the use of such strategies can further boost learners’ confidence.

## Limitations

There are several limitations to consider in this work. First, there are only two items under avoidance, and even though the alpha coefficient was high they did not correlate with the rest of the reading measures. Further, in the validation process no statistically significant differences were detected.

In addition, the number of participants in this work was rather small, and they represented a rather homogenous sample. Thus, future research could examine validity of the scale with a larger number of learners and potentially with a diverse linguistically group and a heterogeneous in performance group to examine sensitivity of the measure and to examine its validity. Of course, this study reports on an exploratory factor analysis, and future research could work to further confirm these findings. Thus, Confirmatory Factor Analysis could examine the factor structure (see Kline, 2015).

Moreover, future research could examine the relationship between self-efficacy and affect and grade level. Potentially as students advance across grade levels and encounter more demanding texts with multisyllabic words that consist of affixes their self-efficacy and affect toward such words declines or it does not as a result of instruction. Future research could also examine students’ performance in relation to quality of instruction and implementation of evidence-based practices.

Finally, in this study we used a Likert scale with a range of 0 to 100. It is possible that the wider range of scores might have affected participants responses. Thus, future research could examine the structure of factors in a 1 to 5 scale to investigate self-efficacy and affect.

## Conclusion

The preceding study sought to examine an instrument designed to capture upper elementary students’ self-efficacy as it relates to reading and spelling bigger, multisyllabic words. The exploratory factor analyses described demonstrate the instrument’s promise and in particular point to the potential existence of independent components (processes and tasks, affect, and avoidance) associated with the overarching construct and further, analyses suggest the varied linkages between these self-efficacy components and reading factors. This study marks an important first step in more deeply understanding the specific affective and motivational factors associated with multisyllabic word reading and spelling.

## Data Availability

The datasets presented in this article are not readily available as analysis is ongoing. Questions regarding the datasets should be directed to the corresponding author.
